# The role of nutrition in integrated programs to control neglected tropical diseases

**DOI:** 10.1186/1741-7015-10-41

**Published:** 2012-04-25

**Authors:** Andrew Hall, Yaobi Zhang, Chad MacArthur, Shawn Baker

**Affiliations:** 1 Centre for Public Health Nutrition, School of Life Sciences, University of Westminster, 115 New Cavendish Street, London W1W 6UW, UK; 2 Helen Keller International, Regional Office for Africa, BP 29.898, Dakar-Yoff, Senegal; 3 Helen Keller International, 352 Park Avenue South, New York, New York 10010, USA

**Keywords:** Neglected tropical diseases, control programs, undernutrition, micronutrients

## Abstract

There are strong and direct relationships between undernutrition and the disease caused by infectious organisms, including the diverse pathogens labeled as neglected tropical diseases (NTDs). Undernutrition increases the risk of infection, the severity of disease and the risk that children will die, while the physical damage, loss of appetite, and host responses during chronic infection can contribute substantially to undernutrition. These relationships are often synergistic. This opinion article examines the role of nutrition in controlling NTDs and makes the point that mass drug treatment - the major strategy currently proposed to control several diseases - is crucial to controlling disease and transmission, but is only the start of the process of physical recovery. Without adequate energy and nutrients to repair damaged tissues or recover lost growth and development, the benefits of treatment may not be evident quickly; the effects of control programs may be not appreciated by beneficiaries; while vulnerability to reinfection and disease may not be reduced. There is substantial potential for nutritional interventions to be added to large-scale programs to deliver drug treatments and thereby contribute, within a broad strategy of public health interventions and behavior change activities, to controlling and preventing NTDs in populations, and to restoring their health.

## Introduction

The fundamental basis of the relationship between an infectious organism and its host is nutritional because the host is the source of all nutrients needed by the organism for maintenance, growth, and reproduction [1]. But the impact of obligate parasites on the nutritional status of a host is not due just to a requirement for nutrients, it is due mainly to the host's responses to infection which lead to an increased metabolic rate, loss of appetite, immune responses, and pathological changes in tissues [2]. Although some of these reactions may be protective to a degree during acute infection, during repeated or chronic infection they can cause the host to become undernourished, especially if the diet is already poor. Undernutrition also increases susceptibility to infection and increases the severity of disease which can lead to a downward spiral of increasing undernutrition and repeated or persistent disease. In this opinion article we argue that breaking out of that spiral requires not just treating disease but treating undernutrition as well, and that nutrition needs to become an important component of integrated programs to control neglected tropical diseases (NTDs).

### Distribution of NTDs and undernutrition

The relatively new classification by the World Health Organization (WHO) of 17 diseases as 'NTDs' includes eight that are caused by at least 23 species of parasitic worms or helminths [3]. The five NTDs of major interest for control efforts listed in Table 1 (intestinal worms, lymphatic filariasis, onchocerciasis, schistosomiasis, and trachoma) have been identified because of the large numbers of people infected, but mainly because there are safe, inexpensive, single-dose drugs to treat them by mass chemotherapy [4]. The hope is to make drugs available free or at a very low cost and deliver them in 'community directed treatment' using a model developed during onchocerciasis control programs [5] or by school teachers to their pupils [6,7]. Mass treatment serves also to decrease transmission by killing larvae or female worms in human hosts, the source of infectious stages. The WHO call this second aim 'preventive chemotherapy' [8] but it does not prevent reinfection with helminths that have infectious stages either already in the environment or developing within intermediate hosts (see Table 1), so repeated treatment is required as well as public health measures and changes in human behavior [9]. This means that chemotherapy alone is not enough to prevent reinfection and does not treat existing undernutrition either, for reasons that will be explained.

**Table 1:** The main neglected tropical diseases for which there are single dose treatments available for disease control.

Group	Generic name or disease	Species	Geographical distribution	Vector or intermediate host	Main drugs available to treat infections	Control measures
Nematode	Large roundworm	*Ascaris lumbricoides*	Worldwide	None, as all species are directly soil-transmitted	Albendazole Mebendazole Oxantel/pyrantel	Sanitation, personal hygiene
	Whipworm	*Trichuris trichiura*				
	Hookworm	*Ancylostoma duodenale*	Worldwide			Sanitation, wearing shoes
	Hookworm	*Necator americanus*				

Nematode	Lymphatic filariasis	*Wuchereria bancrofti*	Worldwide	Mosquitoes	DEC or Ivermectin + Albendazole	Mosquito control
	Lymphatic filariasis	*Brugia malayi*	South Asia			

Nematode	River blindness	*Onchocerca volvulus*	Africa, South America	Black flies	Ivermectin	Black fly control

Trematode	Urinary schistosomiasis	*Schistosoma haematobium*	Africa	Fresh-water snails	Praziquantel	Snail control, sanitation and behavior change
	Intestinal schistosomiasis	*Schistosoma mansoni*	Africa, South America			
	Intestinal schistosomiasis	*Schistosoma japonicum*	Asia			

Bacterium	Trachoma	*Chlamydia trachomatis*	Worldwide	Flies^a^	Azithromycin or Tetracycline	Fly control, personal hygiene

^a^No developmental stage, transmission is passive.

**Figure 1:**
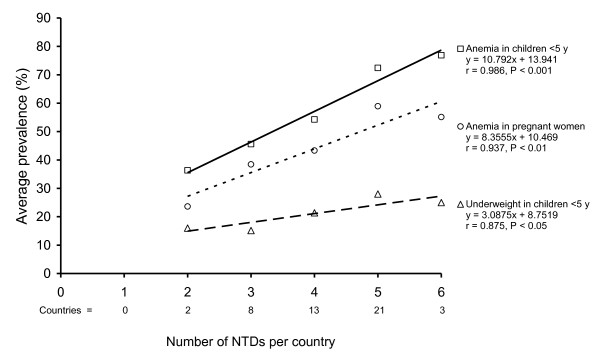
**The association between the number of neglected tropical diseases in 47 countries in Africa **[62]**and the average prevalence of anemia in children aged < 5 years and pregnant women **[71], **and the average prevalence of underweight in children aged < 5 year **[72].

The NTDs listed in Table 1 are widely distributed across the tropics but the major burden of disease occurs in the mainland countries of sub-Saharan Africa where there is transmission of between two and six diseases [10]. These diseases tend to occur amongst the poorest of the poor who are already at risk of diarrheal diseases, respiratory tract infections, malaria, and HIV. But what is sometimes forgotten is that these diseases also occur among people who often suffer from chronic undernutrition as well. For example, Figure 1 shows that there are statistically significant associations between the number of NTDs in 47 countries in Africa and the average prevalence of anemia both in children aged < 5 years and in pregnant women, and with the average prevalence of underweight in young children.

### NTDs and nutrition

Although parasites are dependent on their hosts for their nutritional needs, nutrition is only the first of four inter-related factors that influence the outcome of infection. The second is the virulence of the pathogen, which is often related to the multiplication of microparasites such as bacteria and protozoa, or to the numbers of macroparasites such as helminths acquired, and to where and how they live within the host and cause disease. The third factor is the innate susceptibility of the host to infection, which may be related to the presence of specific receptors on body surfaces or to non-specific defenses against infection such as gastric acidity. The fourth factor is acquired resistance to infection, mediated by cellular and humoral immune responses, both of which are influenced by nutritional status [11].

**Figure 2:**
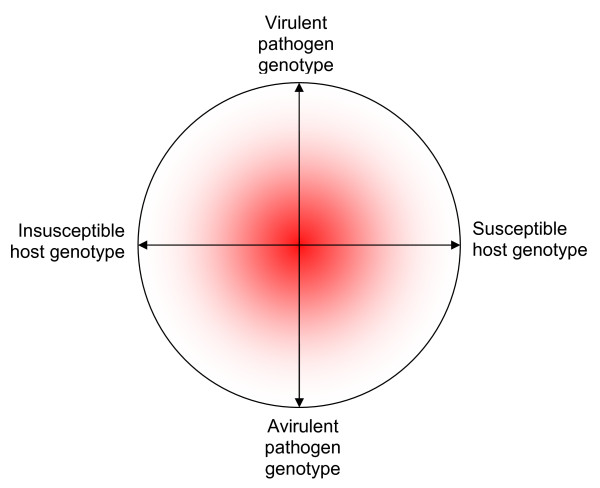
**A conceptual model of the relationship between host susceptibility and pathogen virulence, first proposed by Scrimshaw and colleagues **[12]. The horizontal line represents the natural range in host susceptibility while the vertical line represents the natural range in pathogen virulence. As both are genetically modulated, nutrition is likely to have little or no effect at the extremes: when a pathogen is virulent or avirulent, or when the host is highly susceptible or insusceptible. Nutrition is most likely to have an effect when virulence and susceptibility are intermediate so that other factors may act. This is represented as the shaded area in the centre because the probability that nutrition has an effect on both characteristics is greatest in the middle of both ranges.

Although both virulence and susceptibility are largely genetically mediated, the occurrence or outcome of disease can be influenced by the nutritional status of the host. This is illustrated in Figure 2 based on a theoretical model developed by Scrimshaw and colleagues [12], who proposed that if the organism is extremely virulent or avirulent, then nutrition will probably have no effect. But the main influence of nutrition, indicated by the probability cloud in Figure 2, is in the central ground of moderate virulence and moderate susceptibility where most pathogens and hosts probably lie as a result of natural selection and regression to the mean. The probability that nutrition has an effect at the extremes of virulence and susceptibility is low; the probability is highest when both are moderate.

### Interactions of nutrition and infection

Parasitic organisms and nutrition have separate and combined effects on their host: nutritional status can affect the disease caused by parasitic organisms, parasitic organisms can impair nutritional status, and the two may interact so that their combined effects are synergistic or antagonistic.

A first point to make is that for many NTDs there is a difference between being infected and being diseased. The number of organisms greatly affects the severity of disease and any nutritional effects, particularly for helminths, although the ocular bacterial load of *Chlamydia trachomatis *has also been shown to be related to the severity of trachoma [13], so the relationship is not confined to macroparasites. For this reason the aim of programs to control intestinal helminths and schistosomiasis is usually to reduce worm loads so that the risk of disease is minimal [14], although there is a global goal to eliminate onchocerciasis and lymphatic filariasis.

The evidence for the effect of nutrition on infection and *vice versa*, is hard to obtain. A few longitudinal studies of children have clearly shown that repeated episodes of respiratory and intestinal infections, including worms, are associated with undernutrition and growth failure [15,16]. But as such prospective studies are uncommon and are ethically questionable, much of the evidence comes from cross-sectional surveys. These are difficult to interpret because they suffer from reverse causality bias: did infection cause undernutrition or did undernutrition increase the risk of infection, as undernutrition is both an outcome and a risk factor. For example, if a wasted child is heavily infected with worms did the worms cause the undernutrition or did the undernutrition predispose the child to heavy infection? For these reasons experimental studies of malnourished cells or animals provide the principal means to examine the relationship between nutrition and infection.

**Figure 3:**
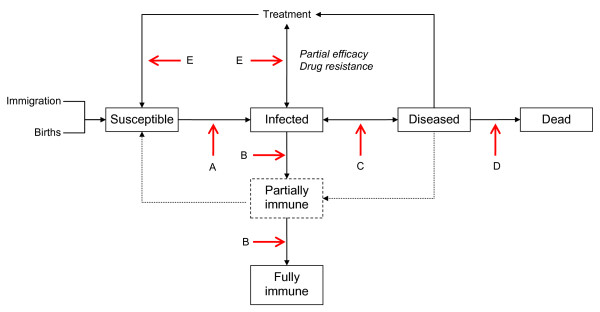
**A diagrammatic flow chart of categories of individuals in the processes of becoming infected, immune, and diseased, and the points at which nutrition may act, shown as arrows with letters indicating points of discussion in the text**.

To help understand how undernutrition may influence the processes of infection and disease, Figure 3 shows a diagrammatic flow chart between five categories of individuals who may be exposed to infectious organisms: susceptible, infected, diseased, immune, or dead. The potential effects of nutrition are marked on Figure 3 with arrows labeled with letters. Potential hosts may be particularly susceptible to infection (Arrow A) because of effects of malnutrition on non-specific defenses such as gastric acidity [17] and the integrity of epithelial surfaces [18]. However a nutrient deficiency may also impair susceptibility: for example, the infectivity of epithelial cells to *C. trachomatis *was lower *in vitro *when grown in a medium containing an iron chelating compound than without, suggesting that iron is required for infection to occur [19].

There is considerable evidence that acquired immune responses (Arrow B), which may be full or partial, are impaired by specific micronutrient deficiencies especially of vitamin A, zinc, and iron [18,20]. Partial immunity is proposed as a reason why some individuals in longitudinal studies do not become heavily reinfected with helminths after treatment [21].

There is sparse evidence that undernourished hosts may become diseased because of a burden of parasites that a well-nourished host would tolerate (Arrow C), but there is evidence for some species that good nutrition can diminish the effects of parasites that might otherwise cause disease. For example a well-nourished host could replace small to moderate blood losses caused by hookworms or schistosomes, so that anemia may not occur [22,23].

There is strong evidence that undernourished hosts are in general at greater risk of dying of infectious diseases than well-nourished hosts (Arrow D), mostly based on analyses of the relationship between underweight and the risk of dying [24,25]. Examples involving NTDs are rare, largely because single infections are rare as polyparasitism is the rule rather than the exception, but also perhaps because NTDs are not a common cause of death. An experimental study in cotton rats of the filarial worm *Litomosoides carinii *showed a greater death rate among protein-deficient animals given the same infectious dose as well-nourished rats [26].

There is a little evidence, also from studies of animals, that drugs may be less effective in undernourished hosts (Arrow E), particularly animals infected with *Schistosoma *spp [27,28], perhaps because the immune responses necessary to kill drug-damaged worms are less effective.

The effects of undernutrition and infection outlined in Figure 3 may not always be for the worse, although generally they are. Undernutrition most often acts synergistically with infection, which means that the effects of both are greater or more serious for a host than would be expected from the combined effect of the two acting independently. But the joint effects may also be antagonistic, in which the combined effect is less than expected, or there may be no interaction. These concepts were developed also by Scrimshaw and colleagues in the same important review, which summarized the findings of 482 papers that had reported the relationship between nutritional deficiencies and infections with bacteria, viruses, protozoa, and helminths [12]. In 67% of studies the relationship was judged to be synergistic, in 19% it was antagonistic and in 13% there was no effect [12]. In nearly 80% of the 94 reported studies of helminths the relationship was judged to be synergistic, so that disease was more severe in malnourished hosts, both animal and human [12]. An update of this review is long overdue.

### The specific effect of NTDs on human nutritional status

The diseases listed in Table 1 cause undernutrition by different mechanisms depending on where the organisms live and how they feed. These have recently been reviewed [29] and are: an increased metabolic rate (fever) as a result of inflammatory responses so that energy and nutrients are used at a greater rate than usual, for example in filarial fever or gut inflammation due to trichuriasis; increased nutrient requirements to meet the needs for immune and pathological responses to worms, larvae, or eggs, for example in filariasis and schistosomiasis; loss of nutrients due to tissue damage, for example due to hookworm or urinary schistosomiasis; malabsorption or maldigestion of nutrients, for example due to intestinal nematode worms; anorexia mediated by inflammatory cytokines released as a response to all infections; and physical debility, reduced activity, or work productivity due to chronic illness, for example due to onchocerciasis, filariasis, or trachoma.

Loss of appetite may make an important contribution to the development of undernutrition and may be exacerbated if the caregiver's response is to withhold food or, for reasons of culture or poverty, give a nutritionally poor diet [30]. Although anorexia may be an important protective response in acute infection, the effects during repeated infection or chronic disease may eventually be harmful [31]. Studies have shown that children's appetite and food intake increase after treating intestinal worms or schistosomiasis [32-34].

Stunted growth and underweight in children are probably among the most important consequences of NTDs, particularly due to intestinal nematode worms [29]. There is also evidence of associations between worms and tests of cognitive function [35-37], but because both are related to poverty and a poor diet, the possibility of confounding is high. A randomized controlled trial in Jamaica of children mostly infected with *Trichuris trichiura *reported effects of anthelmintic treatment on tests of cognition [38], but more such trials are needed.

The loss of blood caused by worms has been estimated to be 0.14-0.26 mL/worm/day for *A. duodenale *[39], 0.02-0.07 mL/worm/day for *N. americanus *[39], and 0.005 mL/worm/day due to *T. trichiura *[40]. The daily losses of blood caused by the eggs of *S. mansoni *and *S. haematobium *cannot be related to the number of worms but have been shown to be as much as 126.0 mL/day [41,42]. There is evidence that perhaps 40% of the iron that passes into the gut due to hookworms may be reabsorbed [43], but blood in urine is lost to the body.

There is no constant linear relationship between worm load and the hemoglobin concentration as the amount of iron in the diet and its absorption efficiency, which typically ranges from 5% to 25% depending on its dietary quality [44], can serve to counterbalance blood loss up to a point, although other micronutrients are required as well for hemopoiesis.

### Recovery of nutritional status after treatment

The main method used to assess whether NTDs have an effect on human health or growth is to undertake randomized trials of treatment, although having untreated controls is now hard to justify. The effects of treatment on most nutritional outcomes is difficult to assess, mainly because drugs kill only worms, they do not provide the nutrients needed for catch-up growth, or for any other consequent deficit for that matter. For example, if hemoglobin is being lost because of hookworms or urinary schistosomiasis then stopping that loss with an anthelmintic drug does not have an effect on the manufacture of hemoglobin in the bone marrow, that rate is mainly dependent on the quality and quantity of the diet to provide nutrients for hemopoiesis. A relative deficiency of even one micronutrient may be rate-limiting, which might explain why some studies of giving iron supplements alone to anemic people after deworming have found no effect on their hemoglobin concentration [45,46]. It also means that studies which compare different anthelmintic treatments in terms of nutritional outcomes such as hemoglobin concentration must be done in the same communities, otherwise differences could be explained by the diet, not by the drug given [47].

As recovery occurs only slowly for chronically undernourished individuals if their diet is not improved, supplementary food or micronutrients are usually required. For example, a study of schoolchildren in Tanzania reported an increase in hemoglobin concentration of 3.6 g/L three months after children were treated with albendazole and praziquantel alone, but the children given supplements of iron and vitamin A in addition showed an increase of 22.1 g/L over the same period [48]. It may also be possible to achieve catch-up growth: therapeutic feeding of Jamaican children treated for *Trichuris *dysentery syndrome found that they grew on average 10.9 cm/year after treatment, more than two standard deviations above the rate expected of children of the same height-for-age [49]. If no nutritional supplements are given then the rate at which body weight or the hemoglobin concentration improves depends on the degree of deficit before treatment and the diet of the people treated, not just on the drug given and its efficacy.

Although supplementary feeding programs are relatively expensive, there is potential to deliver low-cost micronutrient supplements at the same time that people are treated for NTDs, especially to school children [50]. Table 2 shows the current purchase costs of the major single-dose drugs used in NTD control programs and of some common nutritional interventions. The purchase costs are highly dependent on the availability of generic drugs and the number of tablets purchased, while some drugs are now available free. For example, although the nominal cost shown in Table 2 of a tablet of ivermectin is USD 1.50, it has been made available free for treating onchocerciasis since 1987 by the Mectizan Donation Program [51].

**Table 2:** The cost of single dose treatments for some neglected tropical diseases and of several treatments for malnutrition.

Disease or condition	Treatment	Unit	Annual treatment	**Cost in USD of treatments**^**a**^
Intestinal nematode worms	Albendazole	400 mg tab	1-2 tabs	0.02 - 0.04^b^
	Mebendazole	500 mg tab	1-2 tabs	0.03 - 0.06^b^
Schistosomiasis	Praziquantel	600 mg tab	1-3 tabs	0.10 - 0.30
Onchocerciasis	Ivermectin	3 mg tab	1-3 tabs	1.50 - 4.50^c^
Trachoma	Azithromycin	500 mg tab	0.5-2.0 tabs	0.11 - 0.44
Vitamin A deficiency	Retinol	200,000 IU capsule	2 capsules, 1 every 6 months	0.04
Micronutrient deficiencies	Multiple micronutrients^d^	Tablets	24-36 tabs over 3 months	0.39 - 0.59
Thinness and micronutrient deficiencies in schoolchildren	Micronutrient fortified biscuits for school feeding	Approx 75 g (300 kcal) per day	200 days per year	2.66 - 15.92^e^
Wasting and growth faltering in children aged 1-5 years	Ready-to-use therapeutic food	92 g (500 kcal) per sachet	30-60 sachets per child	10.80 - 21.60

^a^Purchase costs of treatments except ivermectin from UNICEF Supply Branch, June 2011.

^b^Now available free from GSK by application through the WHO.

^c^Nominal list price from Merck, but free under Mectizan donation program [51].

^d^Formulation for pregnant women, so typically given two to three times a week to school-age children.

^e^Purchase costs derived from Gelli *et al. *[52] using WFP data; range is for different countries.

As with most treatments, the major financial and economic costs arise from delivering treatments to target populations such as schoolchildren. These costs are hard to standardize or compare for a number of reasons: because methods of costing differ and may not include the costs incurred by schools [52]; because costs differ between countries even if calculated in a standard currency; because of inflationary effects on many costs; and because costs may diminish with economies of scale [52,53]. Nevertheless, delivering an inexpensive treatment such as vitamin A to children in the community may be about 90% of the total cost [54], delivering an equally inexpensive treatment such mebendazole or albendazole to schoolchildren may be around 70% of the total [55], while delivering biscuits to schoolchildren may be about 40% of the total [52]. Although schools offer an existing infrastructure that could minimize the costs of delivering treatments to an often heavily infected and undernourished age group, it should not be expected that teachers will be willing to take on the responsibility of providing health services without adequate training and, perhaps, some reward. There is substantial experience that with sufficient training teachers can administer single dose anthelmintics [6], something that many non-governmental organizations have now been doing as a part of school health programs for almost 20 years. And randomized cluster trials have shown that teachers can effectively deliver weekly iron supplements for 3 months after deworming [56,57]. School-age children are likely to be the greatest beneficiaries from NTD control programs because they have potential to be protected from ill-health and undernutrition and have some capacity to recover before damage is substantial and permanent. They may also, as productive adults, show the quickest returns from investments in NTD control.

But drugs alone are not necessarily 'rapid impact interventions' [10]; they may kill worms quickly, but the recovery of any deficits they have caused will take time, especially if beneficiaries remain in the same nutritionally deprived circumstances [50]. If volunteers in communities that suffer from NTDs are to be convinced to deliver and administer treatments then it could be important for sustainability that there are moderately quick and evident benefits. Part of the success of community directed treatment with ivermectin to treat onchocerciasis may have occurred because treatment alleviates the immediate symptoms of disease, particularly the itching skin of heavily infected adults [58], who tend to be a socially influential group. Studies of mass treatment for lymphatic filariasis in Tanzania indicate that because the drugs did not alleviate the symptoms of disease for adults, the program was not perceived to be useful by some beneficiaries [59]. The value of mass treatment of filariasis lies in protecting children from damage to their lymph ducts, and is not a treatment for elephantiasis. It is also important that beneficiaries understand what they are being treated for and why, especially if they are uninfected or have no disease [59], something that teachers can explain to their pupils.

But most of the pathogens that are the focus of major NTD control efforts do not have specific and identifiable symptoms until disease is severe, while much of the damage to the eyes, skin, lymph ducts, liver, and kidneys is irreversible. If the effects of treating NTDs are to be quickly apparent, particularly to the mothers of children, then supplementary nutrients may be one of the simplest and least expensive interventions that there is to promote recovery after treatment and contribute to NTD control.

### Integrated control programs

Although we are making a case for the value of nutritional supplements after treating NTDs, we recognize that a truly integrated control program needs more than pills. For example, it is possible to treat several NTDs at once where they co-occur [60]; other diseases such as malaria could be controlled at the same time [61-63]; while understanding the social determinants of reinfection [64] and inter-sectoral approaches [65] are potentially useful attributes. But the main point is that drug treatment alone is not enough, as the history of NTD control in countries such as Japan and South Korea has shown; public health measures are required as well [66,67].

**Figure 4:**
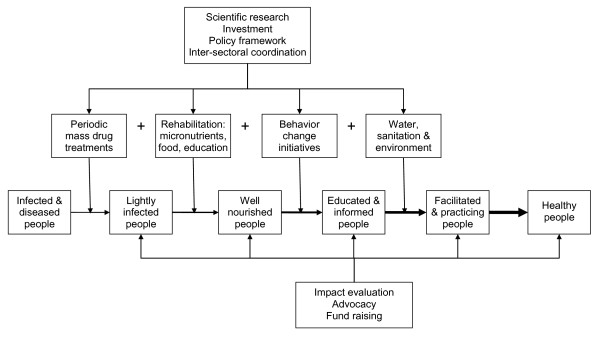
**A conceptual model of the main components of an integrated neglected tropical disease control program**.

Figure 4 illustrates a broad approach in addition to mass drug treatment, including nutritional rehabilitation, behavior change initiatives, and public health measures to prevent reinfection. Mass drug treatment will reduce the burden of disease and diminish sources of reinfection. Rehabilitation is required so that deficits in the health, nutrition, and education of children are recovered as much as possible, and symptoms of disease are alleviated in all age groups, such as entropion in trachoma and skin lesions in elephantiasis. Changes in behavior are needed to help prevent infected people from putting themselves and others at risk of reinfection. Environmental health and sanitation are essential to keep people and their excreta apart and prevent vectors and flies from breeding. And clean water can provide the means to prevent reinfection. The SAFE model of trachoma treatment and control (Surgery, Antibiotics, Face washing, and Environmental measures) offers an example for a single disease [68], but the challenge is to apply broad control measures to several diseases at once.

Figure 4 also makes the point that, as well as inputs such as scientific research, investment, a suitable policy framework, and coordination between different sectors of government, there are also important outputs. Evidence of the impact and benefits of programs will be necessary, particularly for the purposes of advocacy. Nutritional indicators such as anemia and growth are relatively easy to measure as well as being biomedically important outcomes of NTD control [69]. The main technical problem is the need for a control group when evaluating the impact of treatment on children, because their hemoglobin concentration, weight, and height tend to increase with age anyway, albeit more slowly than if they were free of NTDs, and may be affected concurrently by seasonal influences on food supplies and disease. An impact evaluation cannot simply measure children's nutritional status before and after treatment and assume that the treatment was responsible, which is what is sometimes done [70]. But treatments with vitamins and minerals delivered to school-age children are literally vital to build on the benefits of giving drugs to treat NTDs, and together they can help propel the current generation of children and adolescents into a sustainably healthy future.

## Abbreviation

NTD: neglected tropical disease.

## Competing interests

The authors declare that they have no competing interests.

## Authors' contributions

AH was commissioned by Helen Keller International (HKI) to write a review that led to this paper. AH drafted the paper to which all authors contributed and approved the final version.

## Authors' information

AH is a nutritionist and parasitologist who is Visiting Reader in Public Health Nutrition at the University of Westminster, UK. YZ is a physician, parasitologist, and Africa Regional Coordinator for NTD Control for HKI. CM is Director of NTD Control for HKI, based in the USA. SB is a Vice-President of HKI and Regional Director for Africa.

## Pre-publication history

The pre-publication history for this paper can be accessed here:

http://www.biomedcentral.com/1741-7015/10/41/prepub

## References

[B1] Hall A Nutritional aspects of parasitic infection Prog Food Nutr Sci 1985 9 227 256 3914654

[B2] Solomons NW Malnutrition and infection: an update Br J Nutr 2007 Suppl 1 S5 10 17922960 10.1017/S0007114507832879

[B3] World Health Organization Working to overcome the impact of neglected tropical diseases. First WHO report on neglected tropical diseases 2010 Geneva: World Health Organization

[B4] Hotez PJ Molyneux DH Fenwick A Kumaresan J Sachs SE Sachs JD Savioli L Control of neglected tropical diseases N Engl J Med 2007 357 1018 1027 10.1056/NEJMra064142 17804846

[B5] Thylefors B Alleman MM Twum-Danso NA Operational lessons from 20 years of the Mectizan Donation Program for the control of onchocerciasis Trop Med Int Health 2008 13 689 696 10.1111/j.1365-3156.2008.02049.x 18419585

[B6] Partnership for Child Development The cost of large-scale school health programmes which deliver anthelmintics to children in Ghana and Tanzania Acta Trop 1999 73 183 204 10.1016/S0001-706X(99)00028-5 10465058

[B7] Brooker S Marriot H Hall A Adjei S Allan E Maier C Bundy DA Drake LJ Coombes MD Azene G Lansdown RG Wen ST Dzodozmenyo M Cobbinah J Obro N Kihamia CM Issae W Mwanri L Mweta MR Mwaikemwa A Salimu M Ntimbwa P Kiwelu VM Turuka A Nkungu DR Magingo J Partnership for Child Development Community perception of school-based delivery of anthelmintics in Ghana and Tanzania Trop Med Int Health 2001 6 1075 1083 10.1046/j.1365-3156.2001.00806.x 11737845

[B8] World Health Organization Neglected Tropical Diseases. Preventive Chemotherapy and Transmission Control 2006 Geneva: World Health Organization

[B9] Hall A Horton S de Silva N The costs and cost-effectiveness of mass treatment for intestinal nematode worm infections using different treatment thresholds PLoS Neglected Tropical Diseases 2009 3 e402 10.1371/journal.pntd.0000402 19333371 PMC2657832

[B10] Molyneux DH Hotez PJ Fenwick A "Rapid-impact interventions": how a policy of integrated control for Africa's neglected tropical diseases could benefit the poor PLoS Med 2005 2 e336 10.1371/journal.pmed.0020336 16212468 PMC1253619

[B11] Bhaskaram P Micronutrient malnutrition, infection, and immunity: an overview Nutr Rev 2002 60 S40 45 10.1301/00296640260130722 12035857

[B12] Scrimshaw NS Taylor CE Gordon JE Interactions of Nutrition and Infection 1968 Geneva: World Health Organization 4976616

[B13] Solomon AW Holland MJ Burton MJ West SK Alexander ND Aguirre A Massae PA Mkocha H Munoz B Johnson GJ Peeling RW Bailey RL Foster A Mabey DC Strategies for control of trachoma: observational study with quantitative PCR Lancet 2003 362 198 204 10.1016/S0140-6736(03)13909-8 12885481

[B14] World Health Organization Prevention and control of schistosomiasis and soil-transmitted helminthiasis 2002 Geneva: World Health Organization 12592987

[B15] Mata LJ The children of Santa Mara Cauqué 1978 Cambridge, MA: MIT Press

[B16] Checkley W Epstein LD Gilman RH Cabrera L Black RE Effects of acute diarrhea on linear growth in Peruvian children Am J Epidemiol 2003 157 166 175 10.1093/aje/kwf179 12522024

[B17] Martinsen TC Bergh K Waldum HL Gastric juice: a barrier against infectious diseases Basic Clin Pharmacol Toxicol 2005 96 94 102 10.1111/j.1742-7843.2005.pto960202.x 15679471

[B18] Maggini S Wintergerst ES Beveridge S Hornig DH Selected vitamins and trace elements support immune function by strengthening epithelial barriers and cellular and humoral immune responses Br J Nutr 2007 Suppl 1 S29 35 17922955 10.1017/S0007114507832971

[B19] Raulston JE Response of Chlamydia trachomatis serovar E to iron restriction in vitro and evidence for iron-regulated chlamydial proteins Infect Immun 1997 65 4539 4547 9353031 10.1128/iai.65.11.4539-4547.1997 PMC175652

[B20] Wintergerst ES Maggini S Hornig DH Contribution of selected vitamins and trace elements to immune function Ann Nutr Metab 2007 51 301 323 10.1159/000107673 17726308

[B21] Hall A Anwar KS Tomkins AM Intensity of reinfection with *Ascaris lumbricoides *and its implications for parasite control Lancet 1992 339 1253 1257 10.1016/0140-6736(92)91593-W 1349668

[B22] Crompton DW Whitehead RR Hookworm infections and human iron metabolism Parasitology 1993 107 Suppl S137 S145 8115178 10.1017/s0031182000075569

[B23] Torlesse H Hodges M Anthelminthic treatment and haemoglobin concentrations during pregnancy Lancet 2000 356 1083 10.1016/S0140-6736(00)02738-0 11009150

[B24] Pelletier DL Frongillo EAJ Habicht JP Epidemiologic evidence for a potentiating effect of malnutrition on child mortality Am J Public Health 1993 83 1130 10.2105/AJPH.83.8.1130 8342721 PMC1695164

[B25] Black RE Allen LH Bhutta ZA Caulfield LE de Onis M Ezzati M Mathers C Rivera J Maternal and child undernutrition: global and regional exposures and health consequences Lancet 2008 371 243 260 10.1016/S0140-6736(07)61690-0 18207566

[B26] Storey DM The host-parasite relationships in normal and protein-malnourished cotton rats infected with Litomosoides carinii (Nematoda: Filarioidea) Parasitology 1982 85 543 558 10.1017/S0031182000056328 6757846

[B27] Lima SF Souza CT Vieira LQ Coelho PM Protein deficiency impairs the schistosomicidal action of praziquantel Mem Inst Oswaldo Cruz 1998 Suppl 1 271 272 9921368 10.1590/s0074-02761998000700052

[B28] Luttermoser GW DeWitt WB Enhancement of Stibophen (Fuadin(R)) Activity against Schistosoma Mansoni in Mice by Feeding Purified Semi-Synthetic Diets Am J Trop Med Hyg 1961 10 541 546

[B29] Hall A Hewitt G Tuffrey V de Silva N A review and meta-analysis of the impact of intestinal worms on child growth and nutrition Matern Child Nutr 2008 4 118 236 10.1111/j.1740-8709.2007.00127.x 18289159 PMC6860651

[B30] Khan MU Ahmad K Withdrawal of food during diarrhoea: major mechanism of malnutrition following diarrhoea in Bangladesh children J Trop Pediatr 1986 32 57 61 3712529 10.1093/tropej/32.2.57

[B31] Exton MS Infection-induced anorexia: active host defence strategy Appetite 1997 29 369 383 10.1006/appe.1997.0116 9468766

[B32] Hadju V Stephenson L Abadi K Mohammed H Bowman D Parker R Improvements in appetite and growth in helminth-infected schoolboys three and seven weeks after a single dose of pyrantel pamoate Parasitology 1996 113 497 504 10.1017/S0031182000081579 8893536

[B33] Latham MC Stephenson LS Kurz KM Kinoti SN Metrifonate or praziquantel treatment improves physical fitness and appetite of Kenyan schoolboys with *Schistosoma haematobium *and hookworm infections Am J Trop Med Hyg 1990 43 170 2117858 10.4269/ajtmh.1990.43.170

[B34] Stoltzfus RJ Chway HM Montresor A Tielsch JM Jape JK Albonico M Savioli L Low dose daily iron supplementation improves iron status and appetite but not anemia, whereas quarterly anthelminthic treatment improves growth, appetite and anemia in Zanzibari preschool children J Nutr 2004 134 348 356 14747671 10.1093/jn/134.2.348

[B35] Sakti H Nokes C Hertanto WS Hendratno S Hall A Bundy DA Evidence for an association between hookworm infection and cognitive function in Indonesian school children Trop Med Int Health 1999 4 322 334 10.1046/j.1365-3156.1999.00410.x 10402967

[B36] Kvalsvig JD Cooppan RM Connolly KJ The effects of parasite infections on cognitive processes in children Ann Trop Med Parasitol 1991 85 551 568 1809249 10.1080/00034983.1991.11812608

[B37] Ezeamama AE Friedman JF Acosta LP Bellinger DC Langdon GC Manalo DL Olveda RM Kurtis JD McGarvey ST Helminth infection and cognitive impairment among Filipino children Am J Trop Med Hyg 2005 72 540 548 15891127 PMC1382476

[B38] Nokes C Cooper ES Robinson BA Grantham-McGregor SM Sawyer AW Bundy DA Moderate to heavy infections of *Trichuris trichiura *affect cognitive function in Jamaican school children Parasitology 1992 104 539 547 10.1017/S0031182000063800 1641252

[B39] Roche M Layrisse M The nature and causes of "hookworm anemia" Am J Trop Med Hyg 1966 15 1029 1102 5334793

[B40] Layrisse M Roche M Aparcedo L Martínez-Torres C Blood loss due to infection with Trichuris trichiura Am J Trop Med Hyg 1967 16 613 619 6060065 10.4269/ajtmh.1967.16.613

[B41] Farid Z Bassily S Schulert AR Raasch F Zeind AS el Rooby AS Sherif M Blood loss in chronic Schistosoma mansoni infection in Egyptian farmers Trans R Soc Trop Med Hyg 1967 61 621 625 10.1016/0035-9203(67)90124-1 5299185

[B42] Farid Bassily S Schulert AR Zeind AS McConnell E Abdel Wahab MF Urinary blood loss in Schistosoma haematobium infection in Egyptian farmers Trans R Soc Trop Med Hyg 1968 62 496 500 10.1016/0035-9203(68)90132-6 5671535

[B43] Roche M Perez-Gimenez ME Intestinal loss and reabsorption of iron in hookworm infection J Lab Clin Med 1959 54 49 52 13665149

[B44] WHO/FAO Vitamin and mineral requirements in human nutrition 2004 2 Geneva: World Health Organization

[B45] Beasley NM Tomkins AM Hall A Lorri W Kihamia CM Bundy DA The impact of weekly iron supplementation on the iron status and growth of adolescent girls in Tanzania Trop Med Int Health 2000 5 794 799 10.1046/j.1365-3156.2000.00641.x 11123827

[B46] Olsen A Nawiri J Magnussen P Krarup H Friis H Failure of twice-weekly iron supplementation to increase blood haemoglobin and serum ferritin concentrations: results of a randomized controlled trial Ann Trop Med Parasitol 2006 100 251 263 10.1179/136485906X91486 16630383

[B47] Smith JL Brooker S Impact of hookworm infection and deworming on anaemia in non-pregnant populations: a systematic review Trop Med Int Health 2010 15 776 795 10.1111/j.1365-3156.2010.02542.x 20500563 PMC2916221

[B48] Mwanri L Worsley A Ryan P Masika J Supplemental vitamin A improves anemia and growth in anemic school children in Tanzania J Nutr 2000 130 2691 2696 11053508 10.1093/jn/130.11.2691

[B49] Cooper ES Duff EM Howell S Bundy DA 'Catch-up' growth velocities after treatment for *Trichuris *dysentery syndrome Trans R Soc Trop Med Hyg 1995 89 653 10.1016/0035-9203(95)90430-1 8594685

[B50] Hall A Micronutrient supplements for children after deworming Lancet Infectious Diseases 2007 7 297 302 10.1016/S1473-3099(07)70084-1 17376387

[B51] Mectizan Donation Program http://www.mectizan.org

[B52] Gelli A Al-Shaiba N Espejo F The costs and cost-efficiency of providing food through schools in areas of high food insecurity Food Nutr Bull 2009 30 68 76 19445261 10.1177/156482650903000107

[B53] Brooker S Kabatereine NB Fleming F Devlin N Cost and cost-effectiveness of nationwide school-based helminth control in Uganda: intra-country variation and effects of scaling-up Health Policy Plan 2008 23 24 35 18024966 10.1093/heapol/czm041 PMC2637386

[B54] Fiedler JL Sanghvi TG Saunders MK A review of the micronutrient intervention cost literature: program design and policy lessons Int J Health Plann Manage 2008 23 373 397 10.1002/hpm.928 18438981

[B55] Montresor A Gabrielli AF Diarra A Engels D Estimation of the cost of large-scale school deworming programmes with benzimidazoles Trans R Soc Trop Med Hyg 2010 104 129 132 10.1016/j.trstmh.2009.10.007 19926104 PMC5614438

[B56] Hall A Roschnik N Ouattara F Toure I Maiga F Sacko M Moestue H Bendech MA A randomised trial in Mali of the effectiveness of weekly iron supplements given by teachers on the haemoglobin concentrations of schoolchildren Public Health Nutrition 2002 5 413 418 12003652 10.1079/phn2001327

[B57] Roschnik N Parawan A Baylon MA Chua T Hall A Weekly iron supplements given by teachers sustain the haemoglobin concentration of school children in the Philippines Trop Med Int Health 2004 9 904 909 10.1111/j.1365-3156.2004.01279.x 15303996

[B58] Anonymous New light shed on the importance and care of onchocercal skin disease TDR News 1998 55 5 12348563

[B59] Allen T Parker M The "other diseases" of the Millennium Development Goals: rhetoric and reality of free drug distribution to cure the poor's parasites Third World Q 2011 32 91 117 10.1080/01436597.2011.543816 21591302

[B60] Kabatereine NB Malecela M Lado M Zaramba S Amiel O Kolaczinski JH How to (or not to) integrate vertical programmes for the control of major neglected tropical diseases in sub-Saharan Africa PLoS Negl Trop Dis 2010 4 e755 10.1371/journal.pntd.0000755 20614017 PMC2894133

[B61] Gyapong JO Gyapong M Yellu N Anakwah K Amofah G Bockarie M Adjei S Integration of control of neglected tropical diseases into health-care systems: challenges and opportunities Lancet 2010 375 160 165 10.1016/S0140-6736(09)61249-6 20109893

[B62] Hotez PJ Molyneux DH Fenwick A Ottesen E Ehrlich Sachs S Sachs JD Incorporating a rapid-impact package for neglected tropical diseases with programs for HIV/AIDS, tuberculosis, and malaria PLoS Medicine 2006 3 e102 10.1371/journal.pmed.0030102 16435908 PMC1351920

[B63] Temperley M Mueller DH Njagi JK Akhwale W Clarke SE Jukes MC Estambale BB Brooker S Costs and cost-effectiveness of delivering intermittent preventive treatment through schools in western Kenya Malar J 2008 7 196 10.1186/1475-2875-7-196 18826594 PMC2564968

[B64] Spiegel JM Dharamsi S Wasan KM Yassi A Singer B Hotez PJ Hanson C Bundy DA Which new approaches to tackling neglected tropical diseases show promise? PLoS Med 2010 7 e1000255 10.1371/journal.pmed.1000255 20502599 PMC2872649

[B65] Ault SK Intersectoral approaches to neglected diseases Ann N Y Acad Sci 2008 1136 64 69 10.1196/annals.1425.033 18579876

[B66] Hong ST Chai JY Choi MH Huh S Rim HJ Lee SH A successful experience of soil-transmitted helminth control in the Republic of Korea Korean J Parasitol 2006 44 177 185 10.3347/kjp.2006.44.3.177 16969055 PMC2532657

[B67] Kobayashi A Hara T Kajima J Historical aspects for the control of soil-transmitted helminthiases Parasitol Int 2006 Suppl S289 291 16376139 10.1016/j.parint.2005.11.042

[B68] WHO/LSHTM/ITI Trachoma Control. A guide for programme managers 2006 Geneva: World Health Organization

[B69] Bates I McKew S Sarkinfada F Anaemia: a useful indicator of neglected disease burden and control PLoS Med 2007 4 e231 10.1371/journal.pmed.0040231 17696641 PMC1945036

[B70] Kabatereine NB Brooker S Koukounari A Kazibwe F Tukahebwa EM Fleming FM Zhang Y Webster JP Stothard JR Fenwick A Impact of a national helminth control programme on infection and morbidity in Ugandan schoolchildren Bull World Health Organ 2007 85 91 99 10.2471/BLT.06.030353 17308729 PMC2174620

[B71] Benoist de B McLean E Egli I Cogswell M Worldwide prevalence of anaemia 1993-2005 2008 Geneva: World Health Organization 10.1017/S1368980008002401 18498676

[B72] Human development Report 2009. Sheet I-1 Children underweight for age (% under age 5) 2000-2006 http://hdr.undp.org/en/media/HDR_2009_Tables_rev.xls

